# Supplementary material for: A Hotspot Phosphorylation Site on SHP2 Drives Oncoprotein Activation and Drug Resistance
Source: bioRxiv. 2025 Jun 16:2025.06.11.659120. Preprint. [Version 1] doi: 10.1101/2025.06.11.659120 (PMC12262427; doi:10.1101/2025.06.11.659120)
Supplement: Supplement 1 [file media-1.pdf]

Supplementary Table 1: Crystallographic data collection and refinement statistics

	SHP2
PDB entry	9R16
Data processing	

Space group	$P2_1$
Unit cell dimensions<br>a, b, c / Å<br>α, β, γ / °	 45.17, 212.16, 54.92<br>90.0 96.5 90.0
Resolution[a]	34.3-2.63 (3.04-2.63)
Number of reflections<br>Total<br>Unique	 144690 (14234)<br>21059 (2106)
R_{meas}	0.266 (0.834)
R_{pim}	0.101 (0.317)
Mean $I/\sigma I$	5.7 (2.5)
$CC_{1/2}$	0.987 (0.718)
Multiplicity	6.9 (6.8)
Completeness<br>Spherical<br>Ellipsoidal	 69.1 (19.5)<br>89.0 (51.1)
Wilson B-factor / Å^2	54.6
Refinement	
Resolution	27.8-2.63 (2.82-2.63)
R_{work}	0.280 (0.305)
R_{free}	0.304 (0.333)
Number of atoms	8499
Average B-factor	51.0
R.M.S. deviations<br>Bond lengths / Å<br>Bond angles / °	 0.004<br>0.62
Ramachandran plot / %<br>Favored<br>Allowed<br>Outlier	 97.6<br>2.2<br>0.2
Clashscore	0.49

Supplementary Table 2: gRNA and Primer seuences used in this study

	Sequences
PTPN11_gRNA1	GATTACTATGACCTGTATGG
PTPN11_gRNA2	GCGCACTGGTGATGACAAAG
PTPN11_gRNA3	TTACTATGACCTGTATGGAG
Y62D_Forward	CACTGGTGATgatTATGACCTGTATG
Y62D_Reverse	TTCTGAATCTTGATGTGG
Y62F_Forward	CACTGGTGATttcTATGACCTGTATG
Y62F_Reverse	TTCTGAATCTTGATGTGGG
Y542D_Forward	AGGGCACGAAgatACAAATATTAAG
Y542D_Reverse	TTCTGAATCTTGATGTGGG
Y542F_Forward	AGGGCACGAAttcACAAATATTAAG
Y542F_Reverse	TTCCTCTTGCTTTTCTGC
Y580D_Forward	TGCTAGAGTCgatGAAAACGTGG
Y580D_Reverse	CTGTCTTCTCTCATTTCTGC
Y580F_Forward	TGCTAGAGTCttcGAAAACGTGG
Y580F_Reverse	CTGTCTTCTCTCATTTCTG
pLX304_Forward	AACAGCAGAAAAGTTTCAGAGGTAAGCCTATCCCTAACCCTCT
pLX304_Reverse	AACCATCTCCGCGATGTCATTGATCCCGACAGTTAGCCAG
SHP2_Ins_pHAT_Forward	tcatcaccatcaccatcacaacactagtagcgctaccatgATGACATCGCGGAGATGGTT
SHP2_Ins_pHAT_Reverse	gatttaggtgacactatagaatactcaagcttatgcatgcTCATCTGAAACTTTTCTGCTGTTGC
SHP2_Ins_pLX304_Forward	CTGGCTAACTGTCGGGATCAATGACATCGCGGAGATGGTT
SHP2_Ins_pLX304_Reverse	CTGGCTAACTGTCGGGATCAATGACATCGCGGAGATGGTT
pGEX_sequencing_Forward	TGGTAGAACGAAGCGGCG
pGEX_sequencing_Reverse	CGACACCACCACGCTGG
His-tag_Forward	caccaccacGGAATTCCGGGCGGGAGG
His-tag_Reverse	atgatgatgCATGAATACTGTTTCCTGTGTGAAATTGTTATCC

Supplementary Figures:

Supplementary Figure 1:

A

Conservation of SHP2 Y62 across animal species

**Species**	**Common name**	**Sequence**	**Bolded residue**
H. sapiens	*Human*	KIQNTGD**Y**YDLYGGE	Y62
P. troglodytes	*Chimpanzee*	KIQNTGD**Y**YDLYGGE	Y61
M. musculus	*House mouse*	KIQNTGD**Y**YDLYGGE	Y62
M. domestica	*Housefly*	KIQNTGD**Y**YDLYGGE	Y62
G. gallus	*Redfowl*	KIQNTGD**Y**YDLYGGE	Y62
A. carolinensis	*Green Anole*	MIRCQDM**K**YDVGGGE	K62
X. tropicalis	*Western Clawed Frog*	KIQNTGD**Y**YDLYGGE	Y62
D. rerio	*Zebrafish*	KIQNTGD**Y**YDLYGGE	Y62
D. melanogaster	*Fruitfly*	KIQNNGD**F**FDLYGGE	F62
	**SHP1**		
H. sapiens		RIQNSGD**F**YDLYGGE	F60

B

Conservation of Y62 in nSH2 domain in *Homo sapiens*

**Proteins**	**Sequence**	**Phosphosite**	**Frequency**
SHP2	KIQNTGD**Y**YDLYGGE	Y62	2116
RASA1	IIAMCGD**Y**YIGGRRF	Y239	3
YES	RKLDNGG**Y**YITTRAQ	Y222	1816
FYN	RKLDNGG**Y**YITTRAQ	Y213	1819
FGR	RKLDMGG**Y**YITTRVQ	Y208	306
LYN	RSLDNGG**Y**YISPRIT	Y193	787
BLK	RCLDEGG**Y**YISPRIT	Y187	148
SLAP/SLA	FRLPNNW**Y**YISPRLT	Y142	None reported
SH2D1B	IFREKHG**Y**YRIQNSN	Y62	None reported
ITK	TNDNPKR**Y**YVAEKYV	Y305	None reported
BTK	CSTPQSQ**Y**YLAEKHL	Y344	189
TEC	TTTSPKK**Y**YLAEKHA	Y312	None reported

(A) Sequence alignments of SHP2 orthologs across species, and human SHP1. (**B**) Sequence alignments of human SH2 domains with phosphosite and frequency indicated.

Supplementary Figure 2:

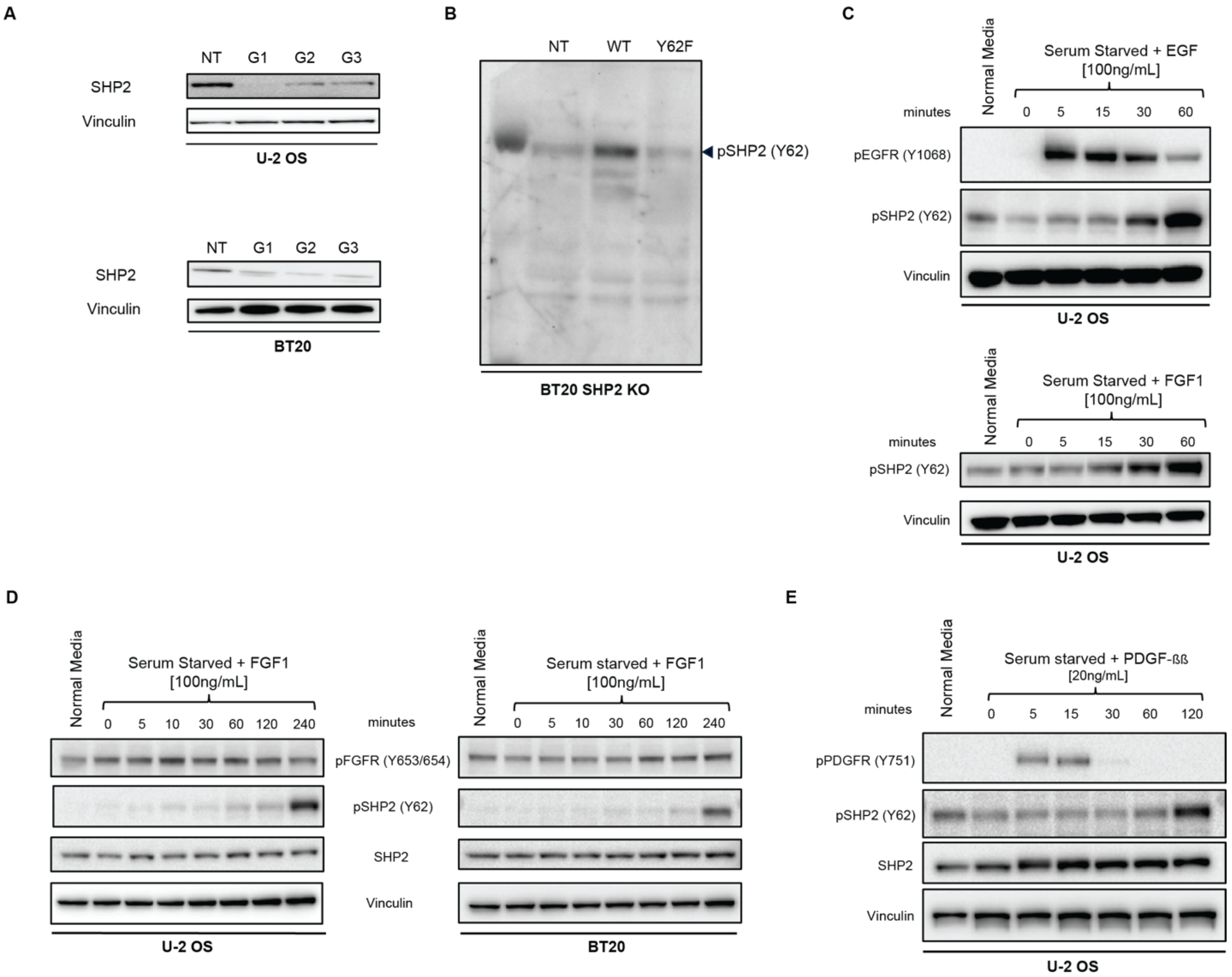

(A) Immunoblot analysis of BT20 and U2-OS cells bearing SHP2 knock out. NT, non-targeting guide RNA. (**B)** Immunoblot analysis of pSHP2 Y62 antibody in BT20 SHP2 knock out cells overexpressing SHP2 WT and Y62F. (**C-E**) Immunoblot analysis of indicated cell lines serum starved (24 hours) then treated with (**C**) EGF **(**100 ng/mL), (**D**) FGF1 (100ng/mL), and (**E**) PDGF-ββ (20ng/mL) for the indicated timepoints.

Supplementary Figure 3:

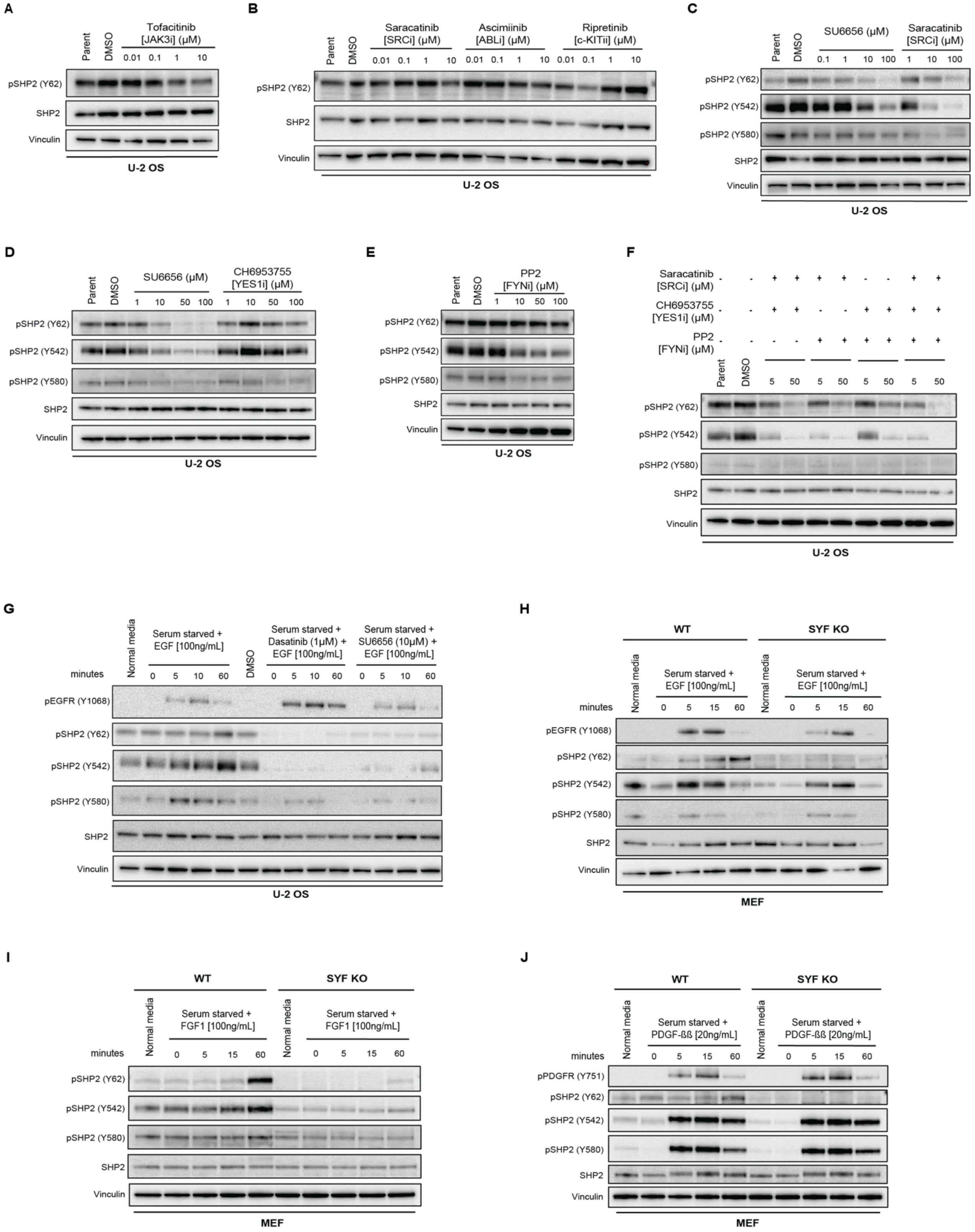

(**A-F**) Immunoblot analysis of U-2 OS cells treated with (**A**) tofacitinb, (**B**) saracatinib, asciminib, and ripretinib, (**C**) SU6656 and saracatinib, (**D**) SU6656 and CH6953755, (**E**) PP2, (**F**) and double and triple combinations of saracatinib, CH6953755 and PP2 at indicated concentrations, and DMSO. (**G**) Immunoblot analysis of U-2 OS cells, serum-starved (24 hours) and treated with dasatinib and SU6656, followed by EGF (100 ng/mL) for indicated timepoints. (**H-J**) Immunoblot analysis of SYF knock out and wildtype MEFs, serum-starved and treated with (**H**) EGF (100 ng/mL), (**I**) FGF1 (100 ng/mL), and (**J**) PDGF-ββ (20 ng/mL).

Supplementary Figure 4:

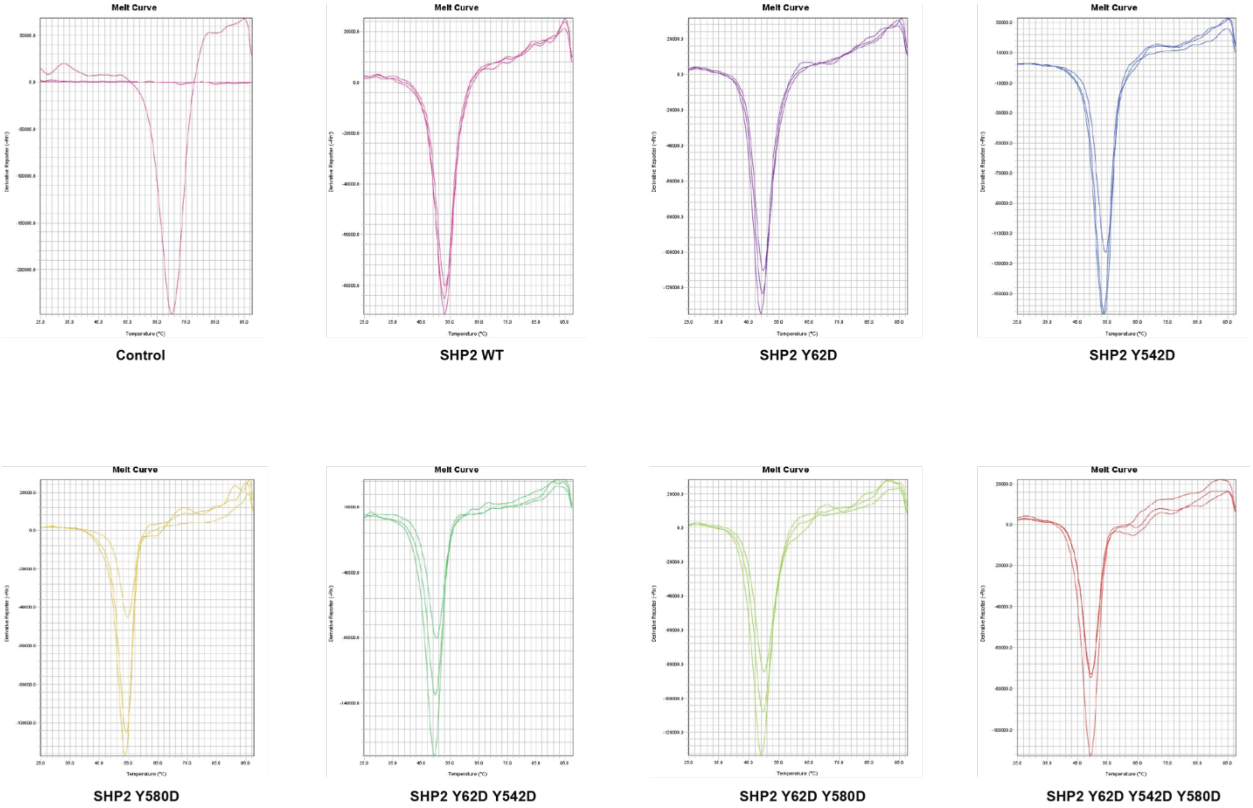

Melting temperatures (T_m) of wild-type and mutant SHP2 proteins by differential scanning fluorimetry (DSF) (n = 3).

Supplementary Figure 5:

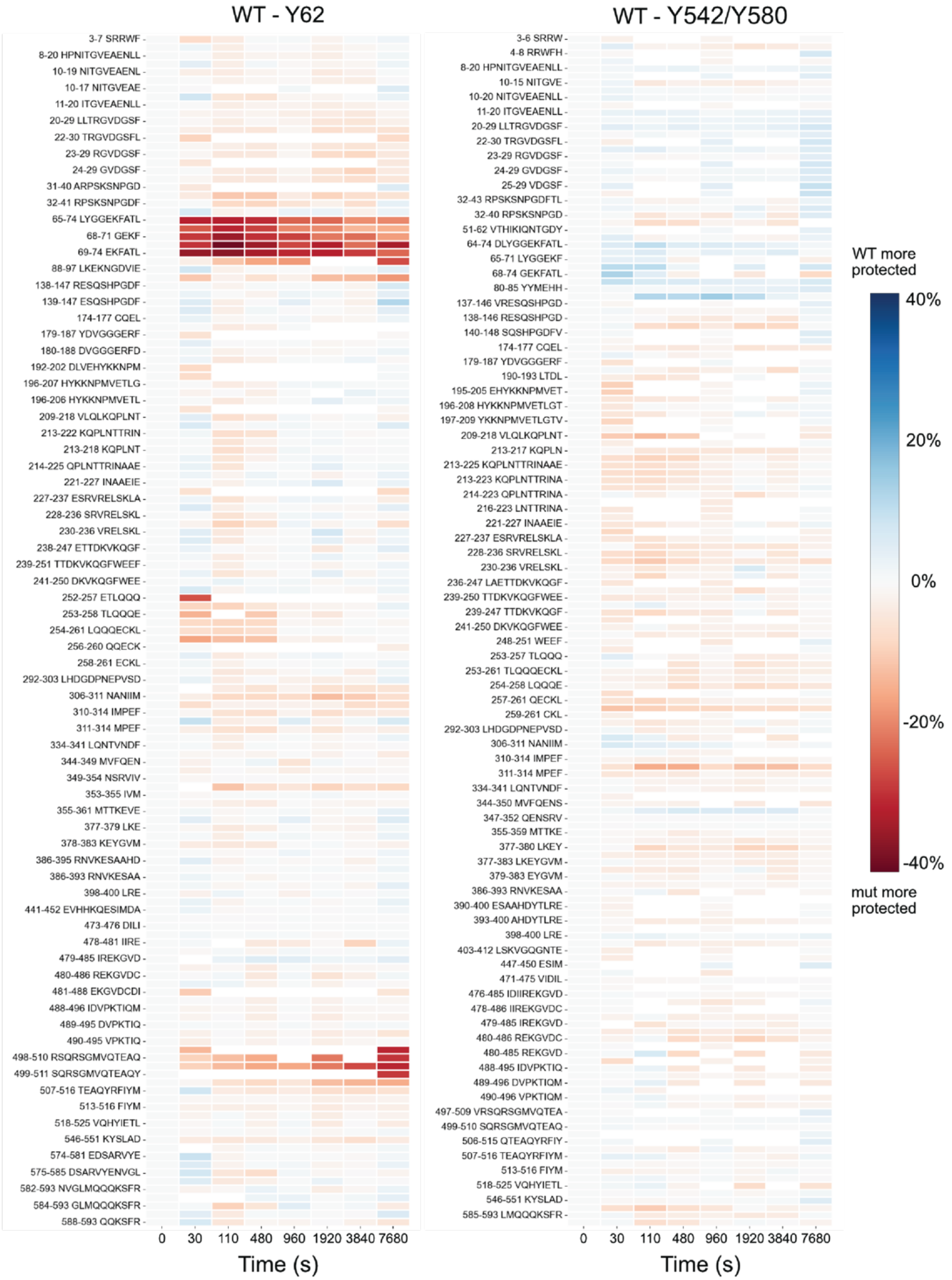

Peptide-level exchange vs time-course heat plots comparing SHP2 WT to the two SHP2 mutants, Y62D and Y542D/Y580D. Plots show peptide length-normalized exchange at each timepoint.
